# Gleaning after the European Headache Federation consensus statement on refractory chronic migraine

**DOI:** 10.1186/1129-2377-15-75

**Published:** 2014-11-13

**Authors:** Paolo Martelletti, Christian Lampl, Michael-Bjorn Russell, Dimos-Dimitrios Mitsikostas

**Affiliations:** 1 Department of Clinical and Molecular Medicine, Sapienza University of Rome, Rome, Italy; 2 Regional Referral Headache Centre, Sant’Andrea Hospital, Rome, Italy; 3 Headache Center Seilerstaette, Department of Neurogeriatric medicine and Remobilisiation, Hospital Barmherzige Schwestern Linz, Lørenskog, Austria; 4 Head and Neck Research Group, Research Center, Akershus University Hospital, Lørenskog, Norway; 5 Institute of Clinical Medicine, Campus Akershus University Hospital, University of Oslo, Nordbyhagen, Norway; 6 Department of Neurology, Naval Hospital, Athens, Greece

## Correspondence/Findings

We appreciated the Comment Letter from the Austrian colleagues referring to the recently published Consensus Statement on clinical definition of refractory Chronic Migraine (rCM), authored by the European Headache Federation (EHF) Expert Group [1,2].

In this Comment Letter [3] the authors present *Chronische Migrane: Therapie, Therapieresistenz und Neuromodulation – Ein Konsensus-Statement*, a consensus statement on CM with and without medication overuse, therapeutic options, with particular focus on patients selection for Occipital Nerve Stimulation (ONS). This article was published in a non-indexed national journal, supported by the device manufacturer [4].

We would like to underline the structural difference existing between the EHF paper and the Austrian one: the first one is finalized to the clinical definition of rCM and the proposal of criteria to be evaluated for a future inclusion of rCM as 3-digit diagnosis of CM in the next ICHD-3 (1.3.1 Refractory Chronic Migraine). The latter mostly targets to patients selection for ONS: “Diagnostic criteria for rCM and guidelines for managing targets patients with rCM and selecting candidates for invasive neuromodulation are crucial issues [4]”. In contrast, the EHF Consensus clearly states “The European Headache Federation felt to develop new consensus criteria that define rCM, particularly for the purposes of controlled clinical trials that involve experimental medication and neuromodulation independently from the non-invasive therapies or the implantable devices [1,5]”.

In particular four points should be addressed:

1. The Notified Body has just removed the CE mark from the only ONS device previously approved for rCM patients [6]. Therefore, any speculation on the definition of rCM criteria useful for ONS selection [4,7] falls exclusively on future randomized controlled trials (RCTs).

2. The semantic debate on EHF criteria “requiring at least 3 different drugs from the following classes” [4] is a misinterpretation of our words: “at least 3 drugs from the following classes” (clearly shown on Table two in our publication) contains 5 classes, 4 of which do not reach 3 items [1]. This fact clearly shows the inconsistency of this criticism. Furthermore, the observation on the minimum dose of prophylactic drugs used is not at all useful in a contest of a definition of refractoriness.

3. The EHF proposed criteria for rCM are defined “inconsistent with respect to MO, since criterion A requires no MO, but recommendations for detoxification are given in the notes”. We think that the wide acceptance of any kind of detoxification procedure guarantees ICHD-3 beta CM diagnosis an uncomplicated purity from acute drugs abuse that might be a confusing factor in the given criteria.

4. The criticism about “laboratory and CSF analyses within the normal range, including CSF pressure”, as reported in the notes at Table two of the EHF Statement, should be seen as a wide evaluation opportunity of many forms of secondary headaches without decontextualize the phrase deleting the term “laboratory”.

We thank the authors of the letter for taking the time to comment our paper, yet this falls partially into our purposes to initiate a European and worldwide discussion on the refractoriness of primary headache disorders, coagulating the various emerging attempts [8].

## Competing interests

The authors declare that they have no competing interests.

## Authors’ contributions

All authors have equally contributed to the manuscript.
